# Use of modeling and simulation to predict the influence of triazole antifungal agents on the pharmacokinetics of zanubrutinib and acalabrutinib

**DOI:** 10.3389/fphar.2022.960186

**Published:** 2022-10-10

**Authors:** Lu Chen, Chao Li, Hao Bai, Lixian Li, Wanyi Chen

**Affiliations:** ^1^ Department of Pharmacy, Chongqing University Cancer Hospital, Chongqing, China; ^2^ Chongqing University, Chongqing, China

**Keywords:** BTK inhibitors, voriconazole, fluconazole, itraconazole, drug-drug interactions, physiologically-based pharmacokinetic

## Abstract

**Background:** Bruton’s tyrosine kinase (BTK) inhibitors are commonly used in the targeted therapy of B-cell malignancies. It is reported that myelosuppression and fungal infections might occur during antitumor therapy of BTK inhibitors, therefore a combination therapy with triazole antifungals is usually required.

**Objective:** To evaluate the influence of different triazoles (voriconazole, fluconazole, itraconazole) on the pharmacokinetics of BTK inhibitors (zanubrutinib, acalabrutinib) and to quantify the drug-drug interactions (DDIs) between them.

**Methods:** The physiologically-based pharmacokinetic (PBPK) models were developed based on pharmacokinetic parameters and physicochemical data using Simcyp^®^ software. These models were validated using clinically observed plasma concentrations data which based on existing published studies. The successfully validated PBPK models were used to evaluate and predict potential DDIs between BTK inhibitors and different triazoles. BTK inhibitors and triazole antifungal agents were simulated by oral administration.

**Results:** Simulated plasma concentration-time profiles of the zanubrutinib, acalabrutinib, voriconazole, fluconazole, and itraconazole are consistent with the clinically observed profiles which based on existing published studies, respectively. The exposures of BTK inhibitors increase by varying degrees when co-administered with different triazole antifungals. At multiple doses regimen, voriconazole, fluconazole and itraconazole may increase the area under plasma concentration-time curve (AUC) of zanubrutinib by 127%, 81%, and 48%, respectively, and may increase the AUC of acalabrutinib by 326%, 119%, and 264%, respectively.

**Conclusion:** The PBPK models sufficiently characterized the pharmacokinetics of BTK inhibitors and triazole antifungals, and were used to predict untested clinical scenarios. Voriconazole exhibited the greatest influence on the exposures of BTK inhibitors. The dosage of zanubrutinib or acalabrutinib need to be reduced when co-administered with moderate CYP3A inhibitors.

## Introduction

Hematologic malignancies are severe hematopoietic diseases which often accompanied by invasive fungal infections (IFIs) (Neofytos et al., 2013; Zeng et al., 2021). This not only due to the malignancies, but also due to the antitumor treatment, such as cytotoxic chemotherapy (Hamalainen et al., 2008), targeted immunotherapies (Lanini et al., 2011), long-term intravenous catheters (Heidenreich et al., 2022), and chemo-radiotherapy (Martino et al., 1997). Hematological malignancies accompanied by IFIs may increase the tumor recurrence and mortality of the patients (Lewis et al., 2013), so it is necessary to start the antifungal treatment as soon as possible.

According to the clinical practice guidelines of Infectious Diseases Society of America (IDSA), triazole antifungal agents are recommended for the prevention and treatment of IFIs, such as voriconazole, fluconazole and itraconazole (Perfect et al., 2010; Pappas et al., 2016; Patterson et al., 2016). Triazole antifungals are mainly metabolized by cytochrome P450 enzymes (CYP450), including CYP2C19, CYP2C9, and CYP3A4 (Bellmann and Smuszkiewicz, 2017), meanwhile they strongly inhibit CYP3A enzymes (Bellmann and Smuszkiewicz, 2017; Han et al., 2021; Ou et al., 2021). In fact, it is difficult to avoid the long-term consolidation therapy for antitumor and antifungal. In this process, the drug-drug interactions (DDIs) may increase the risk of drug toxicities, sub-optimal therapy, and drug resistance.

Over the past decade, with the rapid development of targeted therapy, many tyrosine kinase inhibitors (TKIs) have been approved for the treatment of hematological malignancies. Bruton’s tyrosine kinase (BTK) inhibitors such as zanubrutinib and acalabrutinib are increasingly replacing chemotherapy-based regimens, especially for patients with mantle cell lymphoma (MCL), chronic lymphocytic leukemia (CLL) (Burger, 2019) and small lymphocytic lymphoma (SLL) (Abbas and Wierda, 2021; Tam et al., 2021). According to the pharmacokinetic studies, zanubrutinib and acalabrutinib are mainly metabolized by CYP3A in the liver. When BTK inhibitors are co-administered with triazoles, the exposures of BTK inhibitors tend to increase, which may result in serious adverse effects, such as hematological toxicity, dermatological toxicities and diarrhea (Lipsky and Lamanna, 2020). To the best of our knowledge, at present, only a few reports have suggested the empirical reduction of BTK inhibitors in combination with CYP inhibitors (Hardy-Abeloos et al., 2020; Bruggemann et al., 2022). Therefore, it is essential to evaluate the DDIs between triazoles and BTK inhibitors.

Physiologically-based pharmacokinetic (PBPK) model is a mathematical model that integrated knowledge of physiology, biochemistry and anatomy, in order to simulate the absorption, distribution, metabolism and excretion (ADME) characteristics of drugs in humans (Ellison, 2018). Recently PBPK model has been increasingly accepted by regulatory agencies as a method to inform clinical research strategies. And it has become a useful tool in the simulation of multiple inducers or inhibitors, relevant metabolites, and multiple mechanisms of interaction. Therefore, it has been allowed to predict the complex DDIs involving transporters, enzymes, and multiple interaction mechanisms (Sinha et al., 2014; Sager et al., 2015). The U.S. Food and Drug Administration (FDA) Office of Clinical Pharmacology has been tracking the use of PBPK models in regulatory submissions since 2008. According to 2013 submissions, the models included in regulatory files were most commonly used for DDI (60%), pediatric (21%), and absorption (6%) predictions (Sager et al., 2015). Simcyp (version 20, Certara, Sheffield, United Kingdom), a platform and database for “bottom-up” mechanistic modeling and simulation of the processes of oral absorption, tissue distribution, metabolism and excretion of drugs and drug candidates in healthy and disease populations, is often used to develop PBPK models and to predict the pharmacokinetics and DDIs (Jamei et al., 2009).

In this study, a PBPK model was used to investigate the influence of different triazoles on the pharmacokinetics of BTK inhibitors (zanubrutinib, acalabrutinib) by Simcyp, and the DDIs were quantified to provide a general guidance for the dosage adjustment of BTK inhibitors when co-administered with triazole antifungals.

## Materials and methods

### Physiologically-based pharmacokinetic model development and verification of bruton’s tyrosine kinase inhibitors

**FIGURE 1:**
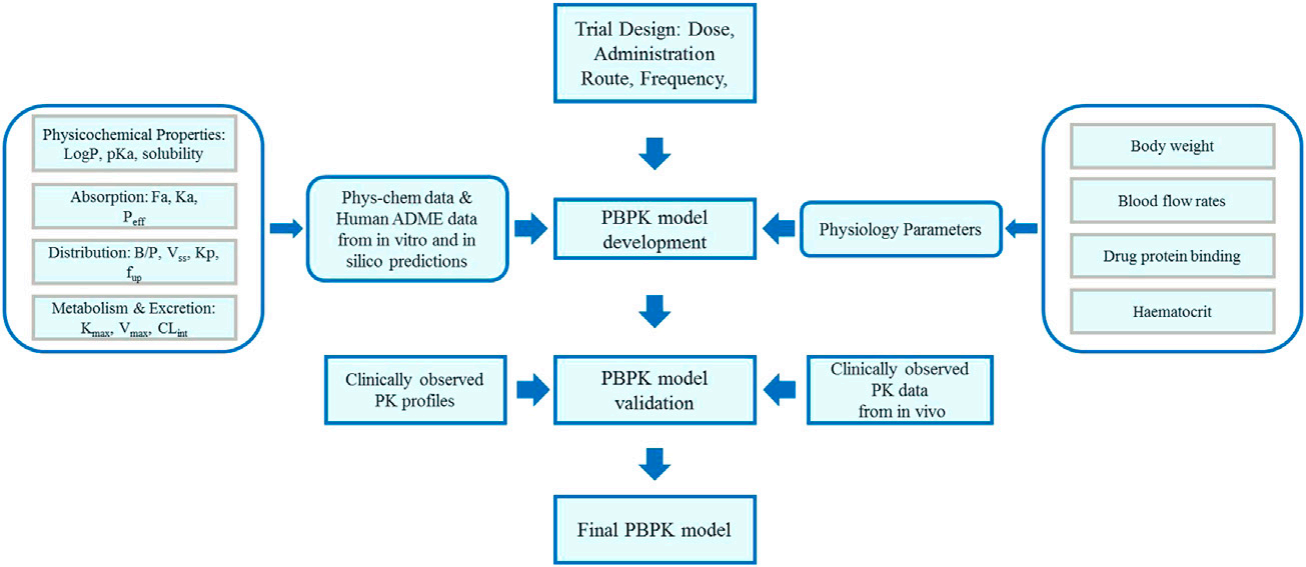
A basic framework of PBPK model development and DDI simulation.

A basic framework of PBPK model development and verification is presented in Figure 1. The developments of zanubrutinib and acalabrutinib PBPK models were based on clinical pharmacokinetic parameters, physicochemical properties data, and *in vitro* experiments parameters. The essential physicochemical properties parameters for the development of PBPK models including molecular weight, the acid dissociation constant (pKa), solubility, octanol/water partition coefficient (logP), fraction unbound in plasma (f_up_), fraction unbound in gut (f_u,gut_), blood-to-plasma concentration ratio (R_bp_), and effective permeability (P_eff_). These physicochemical properties parameters and the corresponding references (Zane and Thakker, 2014; Qi et al., 2017; Li et al., 2018; Zhou et al., 2019; Cai et al., 2020; Li et al., 2020; Wang et al., 2021) are listed in Table 1. The first order absorption model and advanced dissolution, absorption, and metabolism (ADAM) model were used to describe the absorption processes of acalabrutinib and zanubrutinib, respectively. The minimal PBPK model and full PBPK model were used to simulate the distribution processes of acalabrutinib and zanubrutinib, respectively. The selected distribution models are based on published literatures (Zhou et al., 2019; Wang et al., 2021), and the results of model validation showed that the models are reliable and robust. For zanubrutinib, according to the human liver microsome study, the intrinsic clearance value for CYP3A is 120 μL/(minmg); an additional clearance value of 60 µL/(minmg) was inputted to account for non-CYP3A mediated clearance. The renal clearance value is 0.5 L/h (Wang et al., 2021).

**TABLE 1:** Physicochemical property values used for PBPK modeling of zanubrutinib, acalabrutinib, voriconazole, fluconazole and itraconazole.

Parameter	Zanubrutinib Wang et al. (2021)	Acalabrutinib Zhou et al. (2019)	Voriconazole Zane and Thakker. (2014); Qi et al. (2017); Li et al. (2018); Li et al. (2020)	Fluconazole Cai et al. (2020)	Itraconazole Cai et al. (2020)
Base pKa	3.3	3.54, 5.77	1.6	1.76^a^	4.28^a^
Molecular weight (g/mol)	471.55	465.5	349.3	306.3^a^	705.6
Solubility (mg/ml)			3.2	1.39	0.00964
R_bp_	0.804	0.787	1	1^a^	0.58^a^
P_eff_ (×10^−4^cm/s)	0.9	4	3.8	-	0.28
logP	4.2	2.03	1.8	0.2^a^	4.47^a^
P_app,caco-2_ (×10^−6^cm/s)	-	-	-	29.8^a^	-
f_u,gut_ (%)	-	2.6		89^a^	1.6^a^
f_up_ (%)	5.82	2.6	42	89^a^	1.6^a^
CYP3A4 K_m_ (μM)	-	-	-	-	0.004
CYP3A4 V_max_ [pmol/(min·pmol)]	-	-	-	-	0.065
CL_int_ [μL/(min·mg)]	120	9.63μL/min/pmol		-	-
Hepatic CLint [μL/(min·mg)]	-	-	4.3	-	-
Additional clearance HLM [μL/(min·mg)]	60	289.5		-	-
CL_R_ (L/h)	0.5	1.33	0.096	0.86^a^	-
CYP3A4 K_i_	-	-	0.66 μM	10.7 μM^a^	0.001 μM^a^

a From Simcyp Data Management system.

pKa, acid dissociation constant; R_bp_, blood-to-plasma concentration ratio; P_eff_, effective permeability; logP, octanol/water partition coefficient; P_app,caco-2_, apparent permeability of Caco-2 cell line; f_u,gut_, fraction unbound in gut; f_up_, fraction unbound in plasma; K_m_, MichaelisMenten constant; V_max_, maximum rate of metabolism formation; CL_int_, intrinsic clearance; CL_R_, renal clearance.

After the PBPK models were developed, simulations were performed at doses of 80 mg zanubrutinib capsule and 100 mg acalabrutinib capsule which were based on the conventional clinical administration regimens. The time-concentration curves were simulated by PBPK models and the maximum plasma concentration (C_max_) is calculated as the peak concentration in the curve and area under the plasma concentration-time curve (AUC) integrated from 0.00 to t is calculated using log-linear trapezoidal rule in Simcyp. Specifically, Simcyp calculates AUC from 0.00 to t as ${AUC}_{0}^{t}=\sum_{i=1}^{n}{AUC}_{t_{i}}^{t_{i+1}}$ where n is the number of time points in which $t_{1}=0$ and $t_{n+1}=t$ . The rule for ${AUC}_{t_{i}}^{t_{i+1}}$ is as follows. If $C_{i}>C_{i+1}$ , the log-down formula is used to calculate ${AUC}_{t_{i}}^{t_{i+1}}=\frac{C_{i}-C_{i+1}}{\ln\left(\frac{C_{i}}{C_{i+1}}\right)}\times t$ . Otherwise, the linear-up formula is applied as ${AUC}_{t_{i}}^{t_{i+1}}=\frac{C_{i}+C_{i+1}}{2}\times t$ . The developed PBPK models were verified by comparing the simulated plasma concentration curves and pharmacokinetic parameters with corresponding clinically observed plasma concentration curves and pharmacokinetic data in healthy adults which based on existing published studies (Podoll et al., 2019; Ou et al., 2020). The observed data was extracted by applying GetData Graph Digitizer (http://getdata-graph-digitizer.com/). GetData Graph Digitizer is software used to digitize and extract sufficient data (Giang et al., 2019; Shen et al., 2021). The fold-error was used to assess the credibility of the developed PBPK models. The developed PBPK models were considered credible only when the fold-error was less than 2 (Cai et al., 2020). If the observed value is greater than the predicted value, fold-error = observed/predicted; if the observed value is smaller than the predicted value, fold-error = predicted/observed (Fan et al., 2019).

### Physiologically-based pharmacokinetic model development and verification of triazole antifungal agents

The PBPK models developed for triazole antifungal agents were similar to the BTK inhibitors. Voriconazole, fluconazole and itraconazole are all described as inhibitors of CYP3A4 (Bellmann and Smuszkiewicz, 2017). The physicochemical properties parameters used in PBPK models and the corresponding references (Zane and Thakker, 2014; Qi et al., 2017; Li et al., 2018; Zhou et al., 2019; Cai et al., 2020; Li et al., 2020; Wang et al., 2021) are listed in Table 1. The absorption processes of voriconazole, fluconazole and itraconazole were dscribed using the first order absorption models. The distribution processes of voriconazole, fluconazole and itraconazole were dscribed using full PBPK model, minimal PBPK model and minimal PBPK model, respectively. The recombinant enzyme and kinetic parameters [Michaelis-Menten constant (K_m_) and maximum reaction velocity (V_max_)] were used to describe the metabolic process of drugs. The apparent K_m_ and V_max_ values of itraconazole were 0.004 μM and 0.065 pmol/(minpmol) for CYP3A4, respectively. The essential parameters of voriconazole, fluconazole and itraconazole were listed in Table 1. The accuracy of developed PBPK models were verified by comparing the simulated plasma concentration curves and pharmacokinetic parameters with corresponding clinically observed data (Thorpe et al., 1990; Jaruratanasirikul and Sriwiriyajan, 1998; Purkins et al., 2002).

### Drug-drug interactions simulations of bruton’s tyrosine kinase inhibitors and triazole antifungal agents

After the verification, the PBPK model was used to simulate clinical DDI scenarios to quantitatively evaluate the pharmacokinetic changes of zanubrutinib or acalabrutinib when co-administered with triazoles. For the simulation of single dose, all virtual volunteers were given zanubrutinib capsule 160 mg or acalabrutinib capsule 100 mg, combined with 200 mg voriconazole or 200 mg fluconazole or 200 mg itraconazole orally. For the simulation of multiple doses zanubrutinib, the virtual volunteers were given 160 mg zanubrutinib capsule twice daily concomitantly with 200 mg fluconazole once daily for 14 days or 200 mg itraconazole once-daily for 14 days or voriconazole at a loading dose of 400 mg twice-daily (day 1) and a subsequent dose of 200 mg twice-daily (days 2–14). For acalabrutinib group, the virtual volunteers were given 100 mg acalabrutinib capsule twice daily concomitantly with 200 mg fluconazole once daily for 7 days or 200 mg itraconazole once-daily for 7 days or voriconazole at a loading dose of 400 mg twice-daily (day 1) and a subsequent dose of 200 mg twice-daily (days 2–7). The inhibitory potency of triazole antifungals can be measured by the inhibition constant (K_i_) value. The K_i_ values of triazole antifungals were entered into PBPK models to predict the potential DDIs. The K_i_ values of voriconazole, fluconazole and itraconazole were laid in Table 1.

## Results

### Physiologically-based pharmacokinetic model development and verification of bruton’s tyrosine kinase inhibitors and triazole antifungal agents

**FIGURE 2:**
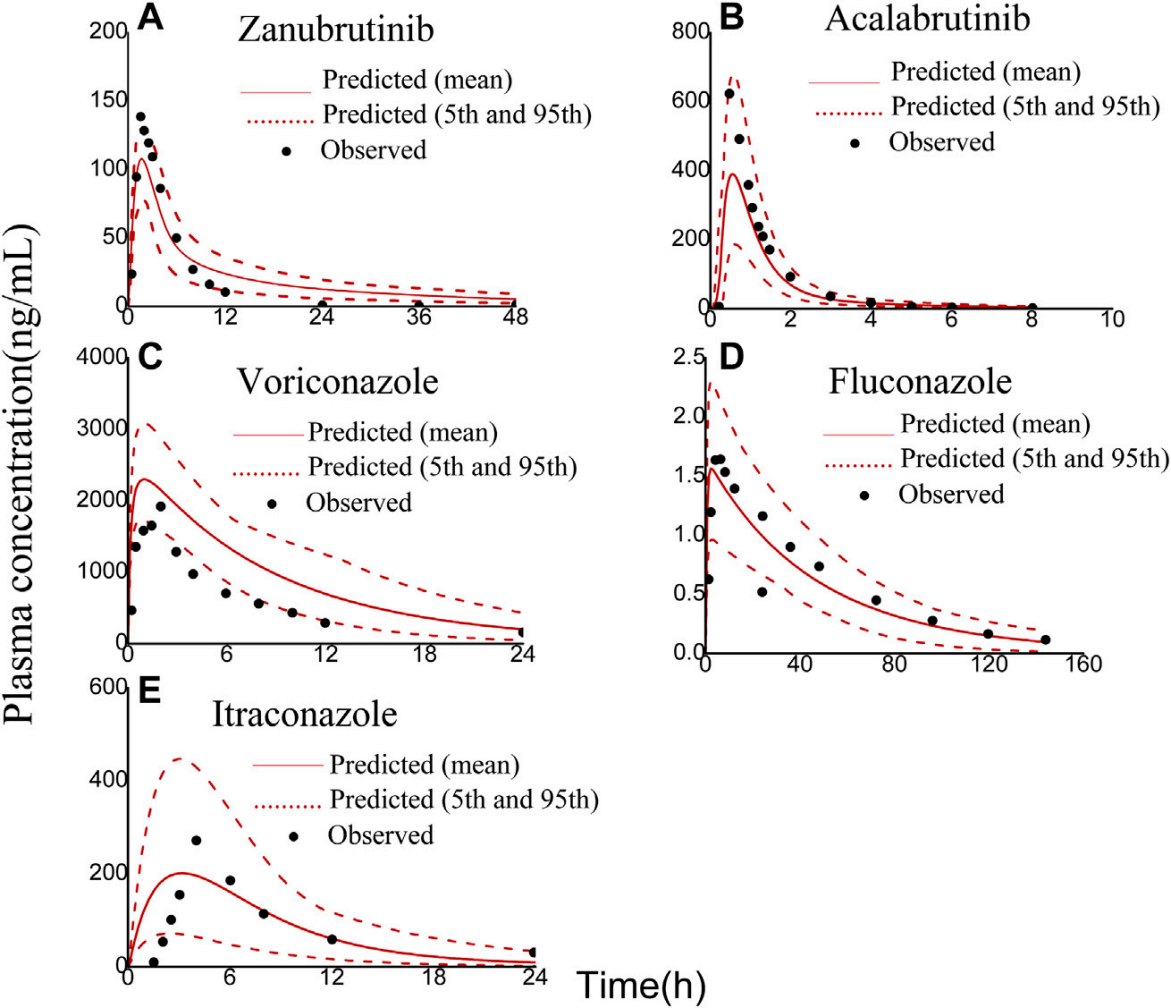
Observed (symbols) and physiologically based pharmacokinetic (PBPK) model simulated (solid lines) plasma concentration-time profiles of zanubrutinib, acalabrutinib, voriconazole, fluconazole, and itraconazole: **(A)** 80 mg zanubrutinib oral; **(B)** 100 mg acalabrutinib oral; **(C)** 200 mg voriconazole oral; **(D)** 100 mg fluconazole oral; **(E)** 200 mg itraconazole oral. The red dashed lines represent the 95th and 5th percentiles of the simulated concentrations.

The robustness of the PBPK models were assessed by comparing predicted with corresponding clinically observed plasma concentration-time profiles and pharmacokinetic parameters (Thorpe et al., 1990; Jaruratanasirikul and Sriwiriyajan, 1998; Purkins et al., 2002; Podoll et al., 2019; Ou et al., 2020). As presented in Figure 2, the predicted plasma concentration curves of zanubrutinib, acalabrutinib, voricoanzole, fluconazole and itraconazole were consistent with the observed curves. Besides, the C_max_ and AUC values were successfully predicted with fold-errors ≤ 2. The C_max_ and AUC values of zanubrutinib, acalabrutinib, voricoanzole, fluconazole and itraconazole and the fold-error values are presented in Table 2. It is obvious that the developed PBPK models are credible.

**TABLE 2:** Observed and predicted PK parameters of zanubrutinib, acalabrutinib, voriconazole, fluconazole and itraconazole.

		C_max_ (ng/ml)	T_max_ (h)	AUC (ng·h/mL)*
Zanubrutinib 80 mg	Observed	162.8	1.5	663
	Predicted	108	1.68	1030
	Fold-error	1.51	1.12	1.55
Acalabrutinib 100 mg	Observed	639	0.5	643
	Predicted	390	0.56	491
	Fold-error	1.64	1.12	1.31
Voriconazole 300 mg	Observed	2360	1.41	12650
	Predicted	2300	0.99	21800
	Fold-error	1.03	1.42	1.72
Fluconazole 100 mg	Observed	1700	4.29	93000
	Predicted	1560	2.49	75200
	Fold-error	1.09	1.72	1.24
Itraconazole 200 mg	Observed	280	4.36	1970
	Predicted	201	3.24	1930
	Fold-error	1.39	1.35	1.02

*AUC_last_ for zanubrutinb, acalabrutinib and voriconazole; AUC_inf_ for fluconazole; AUC_24_ for itraconazole (single dose).

PK, pharmacokinetics; AUC, area under the plasma concentration-time curve; C_max_, maximum plasma concentration; T_max_, time-to-maximum plasma concentration.

### Drug-drug interactions simulations of bruton’s tyrosine kinase inhibitors and triazole antifungal agents

**FIGURE 3:**
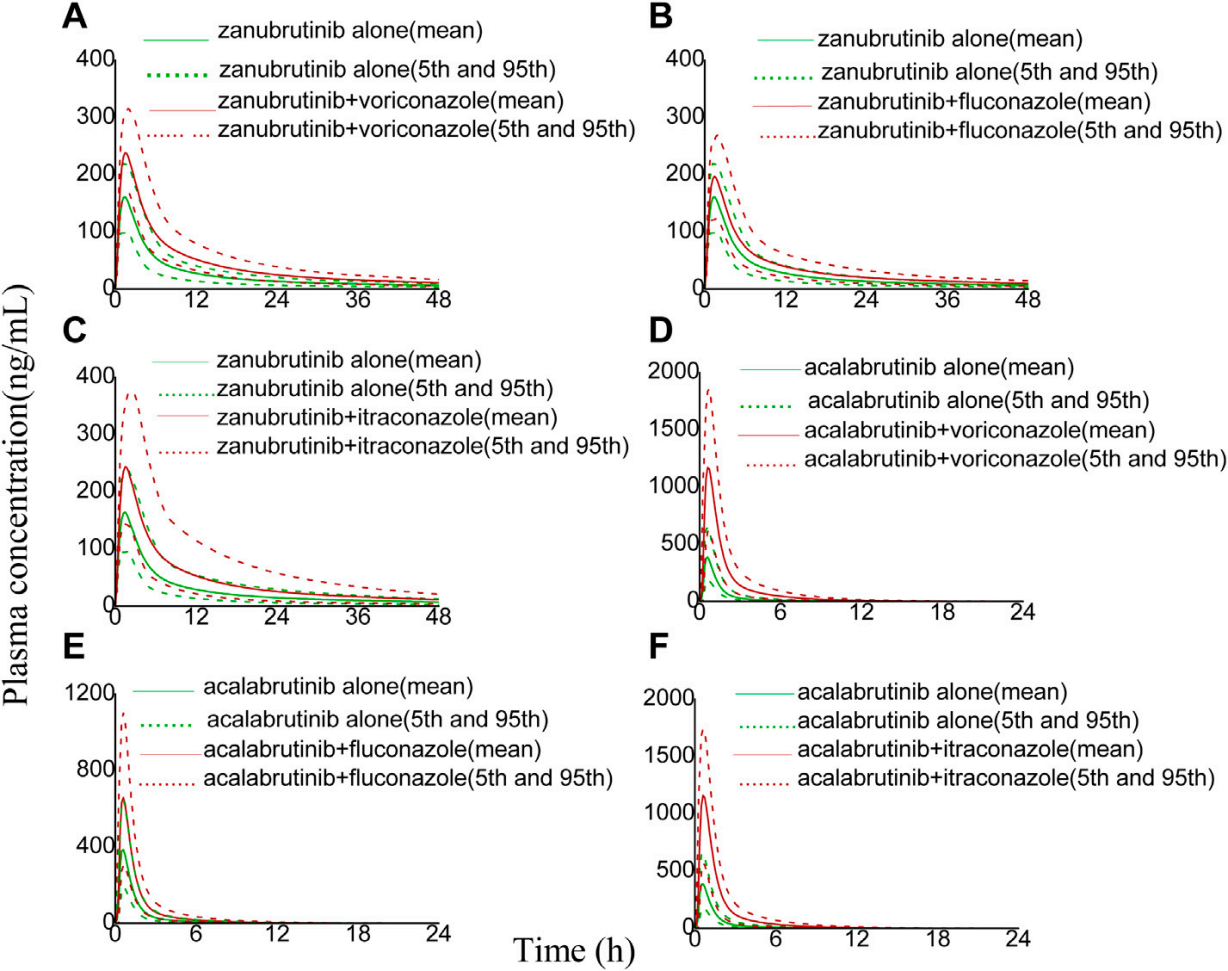
Simulated plasma concentrations of a single-dose zanubrutinib (160 mg) dosed alone or concomitant with **(A)** voriconazole (200 mg), **(B)** fluconazole (200 mg), **(C)** itraconazole (200 mg), and a single-dose acalabrutinib (100 mg) dosed alone or concomitant with **(D)** voriconazole (200 mg), **(E)** fluconazole (200 mg), **(F)** itraconazole (200 mg).

**FIGURE 4:**
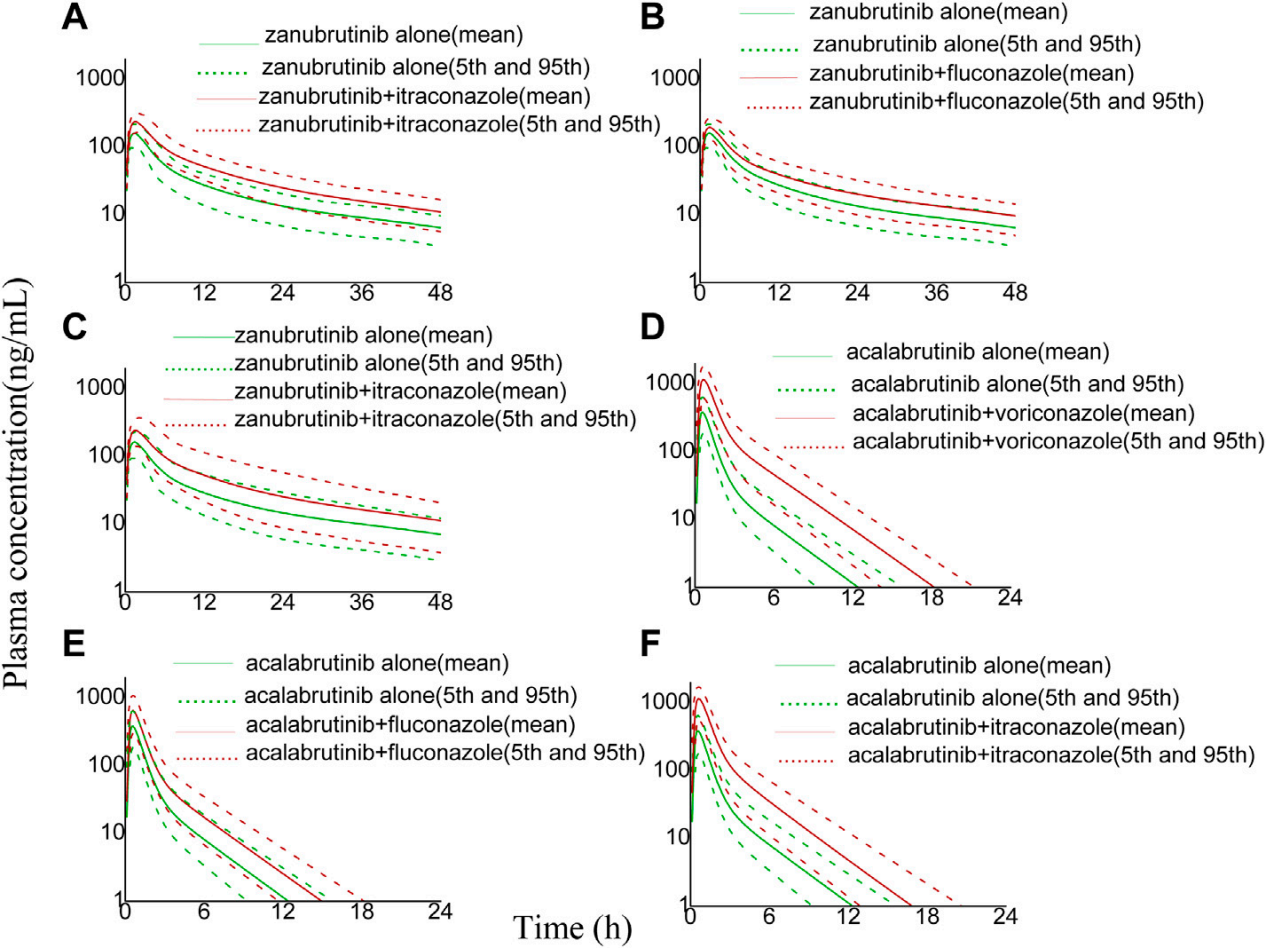
Simulated plasma concentrations (logarithmic concentration axis) of a single-dose zanubrutinib (160 mg) dosed alone or concomitant with **(A)** voriconazole (200 mg), **(B)** fluconazole (200 mg), **(C)** itraconazole (200 mg), and a single-dose acalabrutinib (100 mg) dosed alone or concomitant with **(D)** voriconazole (200 mg), **(E)** fluconazole (200 mg), **(F)** itraconazole (200 mg).

**FIGURE 5:**
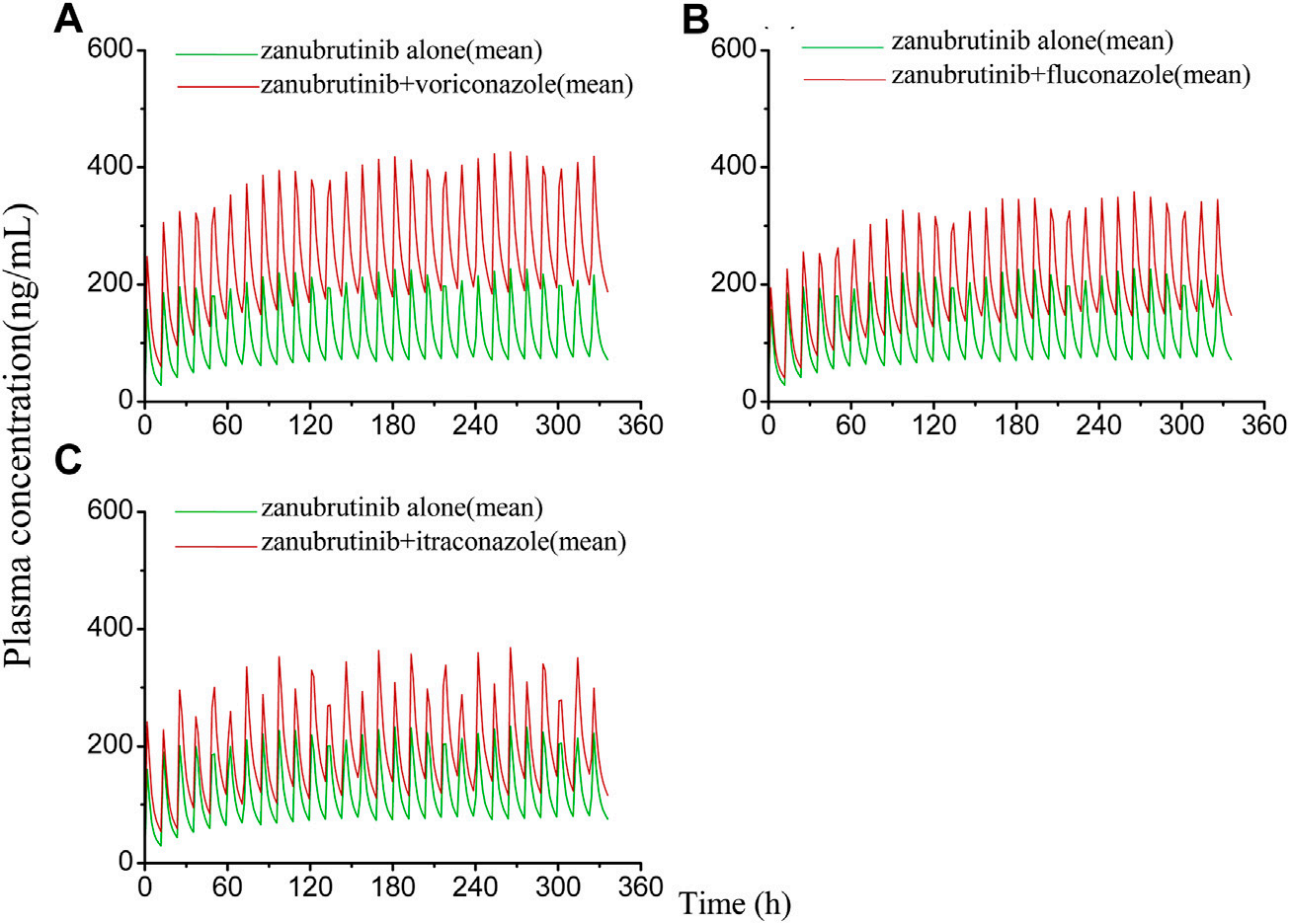
Simulated plasma concentrations of multiple doses (14 days doses) of zanubrutinib (160 mg twice daily) dosed alone or concomitant with **(A)** voriconazole (400 mg twice-daily (day 1) and a subsequent dose of 200 mg twice-daily); **(B)** fluconazole (200 mg once daily); **(C)** itraconazole (200 mg once daily).

**FIGURE 6:**
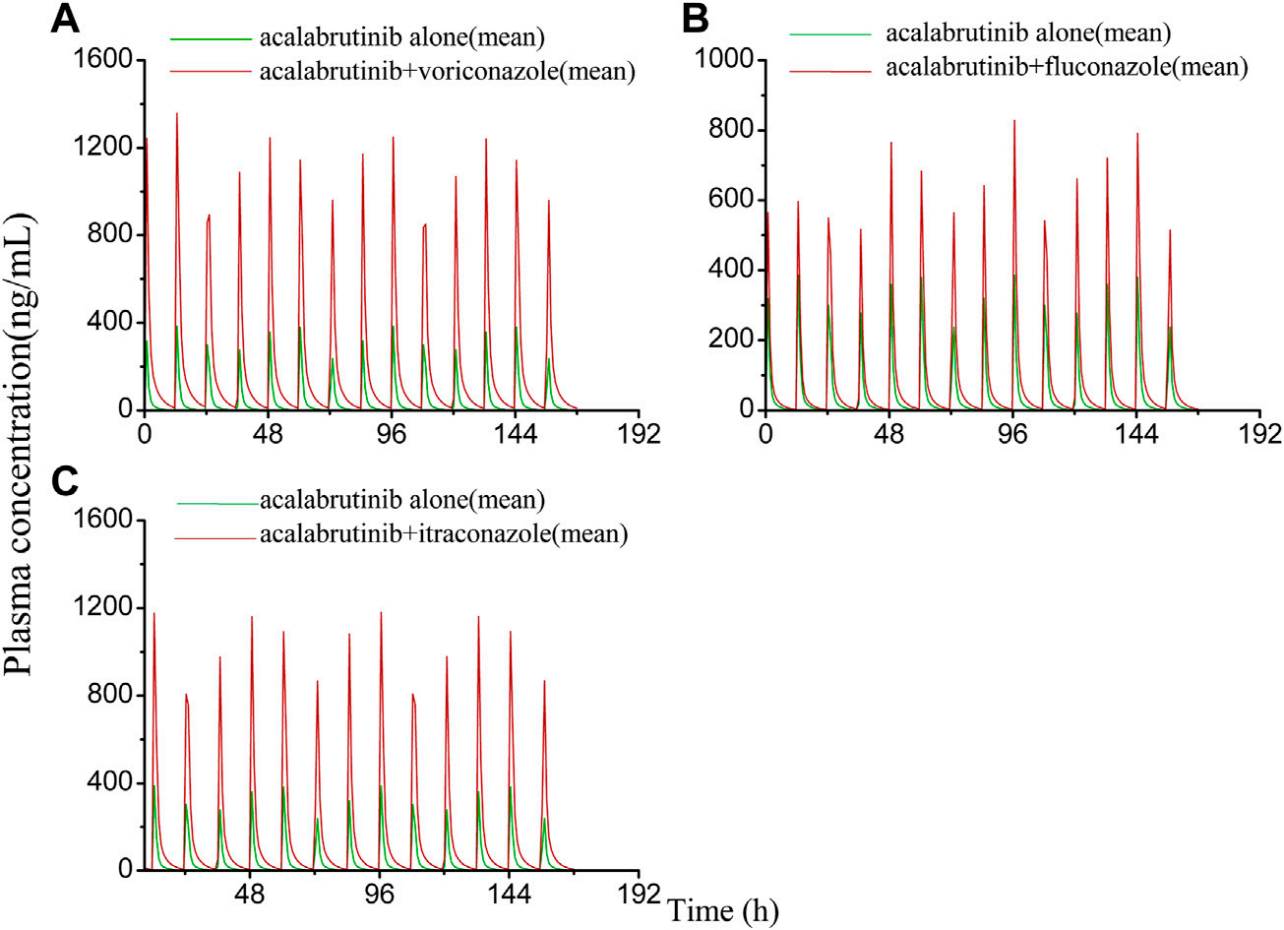
Simulated plasma concentrations of multiple doses (7 days doses) of acalabrutinib (100 mg twice daily) dosed alone or concomitant with **(A)** voriconazole (400 mg twice-daily (day 1) and a subsequent dose of 200 mg twice-daily); **(B)** fluconazole (200 mg once daily); **(C)** itraconazole (200 mg once daily).

The developed PBPK model was applied to predict clinical DDI scenarios for zanubrutinib or acalabrutinib when co-administered with triazole antifungal agents. The simulated DDI results are presented in Table 3, Table 4, Figure 3, Figure 4, Figure 5 and Figure 6. The results indicate that exposures of zanubrutinib and acalabrutinib may increase when co-administered with triazole antifungals. The C_max_ of zanubrutinib increased by 94%, 60%, and 34% and the AUC increased by 127%, 81%, and 48% when co-administered with voriconazole, fluconazole or itraconazole at multiple doses, respectively. The C_max_ of acalabrutinib increased by 220%, 93%, and 200% and the AUC increased by 326%, 119% and 264% when co-administered with voriconazole, fluconazole or itraconazole at multiple doses, respectively. Compared with fluconazole and itraconazole, voriconazole exhibited the greatest influence on exposures of zanubrutinib and acalabrutinib.

**TABLE 3:** Model-predicted PK parameters and ratios of zanubrutinib given alone and with triazoles.

Compound		Parameters		
		C_max_ (ng/ml)	T_max_ (h)	AUC (ng·h/mL)
Zanubrutinib	Alone (single dose)	161	1.44	1290
	DDI with voriconazole (single dose)	238	1.44	2200
	Ratio with voriconazole (single dose)	1.48	1.00	1.71
	Alone (multiple doses)	216	1.92	1580
	DDI with voriconazole (multiple doses)	419	1.92	3580
	Ratio with voriconazole (multiple doses)	1.94	1.00	2.27
	Alone (single dose)	161	1.44	1290
	DDI with fluconazole (single dose)	197	1.44	1760
	Ratio with fluconazole (single dose)	1.22	1.00	1.36
	Alone (multiple doses)	216	1.92	1580
	DDI with fluconazole (multiple doses)	345	1.92	2860
	Ratio with fluconazole (multiple doses)	1.60	1.00	1.81
	Alone (single dose)	164	1.44	1350
	DDI with itraconazole (single dose)	243	1.44	2250
	Ratio with itraconazole (single dose)	1.48	1.00	1.67
	Alone (multiple doses)	222	1.92	1640
	DDI with itraconazole (multiple doses)	299	1.92	2430
	Ratio with itraconazole (multiple doses)	1.34	1.00	1.48

PK, pharmacokinetics; DDI, drug-drug interaction.

**TABLE 4:** Model-predicted PK parameters and ratios of acalabrutinib given alone and with triazoles.

Compound		Parameters		
		C_max_ (ng/ml)	T_max_ (h)	AUC (ng·h/mL)
Acalabrutinib	Alone (single dose)	385	0.6	513
	DDI with voriconazole (single dose)	1170	0.6	1930
	Ratio with voriconazole (single dose)	3.04	1.00	3.76
	Alone (multiple doses)	402	1.08	513
	DDI with voriconazole (multiple doses)	1286	1.08	2184
	Ratio with voriconazole (multiple doses)	3.20	1.00	4.26
	Alone (single dose)	385	0.6	513
	DDI with fluconazole (single dose)	658	0.6	937
	Ratio with fluconazole (single dose)	1.71	1.00	1.83
	Alone (multiple doses)	402	1.08	513
	DDI with fluconazole (multiple doses)	776	1.08	1124
	Ratio with fluconazole (multiple doses)	1.93	1.00	2.19
	Alone (single dose)	387	0.6	512
	DDI with itraconazole (single dose)	1160	0.6	1790
	Ratio with itraconazole (single dose)	3.00	1.00	3.50
	Alone (multiple doses)	404	1.08	513
	DDI with itraconazole (multiple doses)	1213	1.08	1865
	Ratio with itraconazole (multiple doses)	3.00	1.00	3.64

PK, pharmacokinetics; DDI, drug-drug interaction.

## Discussion

The results of the DDI simulations showed that the pharmacokinetic exposures of zanubrutinib and acalabruitnib increased to varying degrees when combined with voriconazole, fluconazole, or itraconazole, respectively. In brief, compared with taking zanubrutinib alone, the AUC of zanubrutinib increased by 127%, 81%, and 48% when combined with voriconazole, fluconazole or itraconazole at multiple doses, respectively. Furthermore, compared with taking acalabrutinib alone, the AUC of acalabrutinib increased by 326%, 119%, and 264% when combined with voriconazole, fluconazole or itraconazole at multiple doses, respectively.

According to the results above, co-administered of BTK inhibitors and triazoles will increase the pharmacokinetic exposures of BTK inhibitors, and among the three triazoles, voriconazole exhibited the most significant effect on the pharmacokinetic exposures of zanubrutinib and acalabruitnib. Nonetheless, the degree of elevation was markedly different between zanubrutinib and acalatinib, especially co-administered with voriconazole and itraconazole. The reason may be related to the fact that zanubrutinib can decrease the systemic exposure of CYP3A and CYP2C19 substrates (Ou et al., 2021). Voriconazole, which happens to be a substrate for CYP2C19, CYP2C9 and CYP3A4, and itraconazole is a substrate for CYP3A4 (Bellmann and Smuszkiewicz, 2017). Therefore, zanubrutinib decreased the systemic exposures of voriconazole and itraconazole, resulting in less inhibitory effects on zanubrutinib caused by voriconazole and itraconazole compared with acalabrutinib. Whereas fluconazole’s metabolic pathways are not qualitatively or quantitatively significant, and its main route of elimination is renal excretion (Debruyne and Ryckelynck, 1993), which will not be influenced by zanubrutinib and acalabruitnib, so both of the pharmacokinetic exposures increased in similar degree.

Therapeutic drug monitoring (TDM) is the clinical practice of measuring drugs at specified time intervals to support individualized PK-based dose adjustments, thus maintaining consistent concentrations in patient’s blood, reducing regimen-related toxicities and improving treatment efficacy. TDM has been shown its advantage in optimization the dosing of voriconazole (Ashbee et al., 2014), vancomycin (Pai et al., 2014), valproic acid (Johannessen Landmark et al., 2020), cyclosporine (Jorga et al., 2004) and so on. Moreover, the exposure-response and/or exposure-toxicity relationships of several oral targeted antineoplastic drugs have been established, and TDM has been proven to be practical for individualized dosing of imatinib, sunitinib, abiraterone, everolimus, etc., (Verheijen et al., 2017; Mueller-Schoell et al., 2021). Even though there has not any recommendation for TDM of the BTK inhibitors to date, TDM can still be conducted to clarify the DDIs between BTK inhibitors and triazole antifungal agents, so as to guide individualized dosing, optimize therapy and prevent toxicity. Overall, our study indicated that in order to avoid the increased concentration of BTK inhibitors, we should reduce the dosage of BTK inhibitors when co-administered with triazoles, especially voriconazole.

Although the PBPK model is well-established, reasonably refined and validated, limitations still exist in the present study. Firstly, genetic polymorphisms of CYP3A4 may alter the metabolic enzyme activities of zanubrutinib and acalabrutinib. The inhibitory potency also varies among different variants when co-administered with a CYP inhibitor (Han et al., 2021). Secondly, the DDIs between zanubrutinib, acalabrutinib and triazoles were predicted in healthy subjects in our study. However, the enzyme activity of CYP3A4 may be different in disease state such as CLL, SLL, and MCL (Gao et al., 2022). Therefore, the DDIs between zanubrutinib, acalabrutinib and triazoles in patients with hematologic malignancies need to be studied in further research.

## Conclusion

In conclusion, the developed and validated PBPK models were successfully used to predict the DDIs between zanubrutinib, acalabrutinib and different triazoles. Compared with taking zanubrutinib or acalabrutinib alone, the pharmacokinetic exposures of zanubrutinib and acalabruitnib increased to varying degrees when co-administered with voriconazole, fluconazole, or itraconazole, respectively. The dosage of zanubrutinib and acalabrutinib need to be reduced when co-administered with triazole antifungal agents.

## Data Availability

The original contributions presented in the study are included in the article/supplementary material, further inquiries can be directed to the corresponding author.
